# A review of UHMWPE wear-induced osteolysis: the role for early detection of the immune response

**DOI:** 10.1038/boneres.2016.14

**Published:** 2016-07-12

**Authors:** Adrese M Kandahari, Xinlin Yang, Kevin A Laroche, Abhijit S Dighe, Dongfeng Pan, Quanjun Cui

**Affiliations:** 1 Department of Orthopaedic Surgery, University of Virginia, Charlottesville, VA, USA; 2 Department of Radiology and Medical Imaging, University of Virginia, Charlottesville, VA, USA

## Abstract

In a world where increasing joint arthroplasties are being performed on increasingly younger patients, osteolysis as the leading cause of failure after total joint arthroplasty (TJA) has gained considerable attention. Ultra-high molecular weight polyethylene wear-induced osteolysis is the process by which prosthetic debris mechanically released from the surface of prosthetic joints induces an immune response that favors bone catabolism, resulting in loosening of prostheses with eventual failure or fracture. The immune response initiated is innate in that it is nonspecific and self-propagating, with monocytic cells and osteoclasts being the main effectors. To date, detecting disease early enough to implement effective intervention without unwanted systemic side effects has been a major barrier. These barriers can be overcome using newer *in vivo* imaging techniques and modules linked with fluorescence and/or chemotherapies. We discuss the pathogenesis of osteolysis, and provide discussion of the challenges with imaging and therapeutics. We describe a positron emission tomography imaging cinnamoyl-Phe-(D)-Leu-Phe-(D)-Leu-Phe-Lys module, specific to macrophages, which holds promise in early detection of disease and localization of treatment. Further research and increased collaboration among therapeutic and three-dimensional imaging researchers are essential in realizing a solution to clinical osteolysis in TJA.

## Introduction

Total joint arthroplasty (TJA) is often considered one of the success stories of modern medicine, as it provides reliable long-term improvement in patient function, pain, and quality of life.^1–8^ Aseptic loosening (AL) by periprosthetic osteolysis is the leading cause of failure after TJA^4–6,9,10^ with reported incidence rates ranging from 10% to 70%.^4,5,11,12^ The incidence of TJA is increasing, with over four million primary total hip/knee arthroplasties (THA, TKA) projected annually by 2030 in the United States and revision rates expected to double even earlier.^13^ Patients under 65 years of age currently comprise over 40% of the population undergoing THA or TKA, with incidence among this age group expected to increase.^7,14^ As more TJA is performed on increasingly younger patients, rates of osteolysis-induced AL will intensify, as young age is a known risk factor for osteolysis, TJA failure, and revision surgery.^4,11,15^ Compared with primary THA or TKA, revision surgery has been shown to be costlier, technically more difficult, and associated with higher rates of complications and inferior outcomes.^8,15–19^ Considering this evidence, the vast impact TJA will have on US healthcare and economy in the near future quickly becomes evident. Although understanding of the mechanisms underlying osteolysis-induced AL has substantially improved in recent decades, barriers in the implementation of effective intervention obviating the need for revision surgery or minimizing its technical and financial demands still exist. These barriers revolve around difficulties in localization: early detection as osteolysis is often asymptomatic or undiagnosed until the need for revision surgery,^4,8,17,20^ and therapy that is both effective and specific enough as to avoid intolerable or undesirable systemic side effects.^5,8,21,22^

Periprosthetic osteolysis is the process by which biological or mechanical forces initiate a local immune response in periprosthetic tissue that eventually results in implant loosening and failure. Although this could be due to a number of factors such as infection, wear-induced osteolysis is induced by wear particles mechanically released at the articulating surface of prostheses over time. The material and character of the prosthesis implanted have implications on this process^4–7,9,15,22,23^ with research focusing on developing prostheses with decreased rates of wear and therefore osteolysis and AL. Ultra-high molecular weight polyethylene (UHMWPE) is a plastic bearing commonly utilized in TJA, as it is durable and has low antigenicity and well-established clinical success.^7,15^ Recently, highly cross-linked UHMWPE has come into use, proven *in vitro* to decrease wear by up to 92%^24^ with promising short-term *in vivo* results.^15,25–27^ Despite these advances, long-term data are lacking and osteolysis will continue to be a burden for patients after TJA, especially as patients undergoing these procedures become younger and younger. Recent research has characterized an immune response dominated by cells of monocytic or osteoclastogenic lineage when challenged with UHMWPE wear particles;^5,6,8,22,28–31^ the current study explores that response: its players, progression, and future direction for timely detection and intervention.

## UHMWPE wear debris: an inciting factor

Prostheses utilized in TJA serve to mimic the function of the natural joint and therefore face similar mechanical and chemical challenges: providing a smooth, durable, sliding surface that can afford sufficient and stable movement without pain over time. To attain the functional success of hyaline cartilage *in vivo*, prostheses must then balance the elements of friction, lubrication, and wear.^4,32^ Wear refers to the loss of prosthetic material from the interface of articulating surfaces, and can be due to abrasion, adhesion, or fatigue.^32^ This debris of wear particles deposits in periprosthetic tissue, eliciting a chain reaction that results in the phenomenon of osteolysis.

As the works of Revell *et al.*^33^ and Mirra *et al.*^34^ first reported the associations between excessive polyethylene (PE) debris and loss of bone tissue and component loosening in 1978 and 1982, respectively; the presence of periprosthetic wear debris has been demonstrated to coincide with the diagnosis of osteolysis in countless models.^8,9,28,29,31^ A dose-dependent response has been described between the two: as UHMWPE wear particles are increasingly generated, risk for osteolysis intensifies.^35,36^ At the heart of this dose-dependent response thrives the macrophage with histographic cell counts positively correlating with amount of PE debris and bone resorption.^28,29,31,37^ It logically follows that UHMWPE wear debris is capable of initiating an immune response mediated by macrophages, and this principle has led to the study of particulates and development of PE with unique properties in an attempt to circumvent this response. Schmalzried *et al.*^37^ examined histological specimens from areas of bone resorption in 34 hips with radiographic evidence of periprosthetic bone loss and universally detected PE debris-laden macrophages regardless of pattern of bone loss, anatomic origin of specimen, or use of cement. PE particles were mostly intracellular and <10 μm in length, with macrophage infiltration positively correlating with the amount of PE debris and degree of bone resorption observed.

Though highly variable, UHMWPE wear particulates generally range from 0.1 to 1.0 μm (average 0.5 μm) in length when recovered from the hip, but average ~280 μm in length when recovered from the knee.^6,7,31^ Particulates in the range of 0.1–1.0 μm are thought to be the most biologically active, with reported critical lengths of roughly 0.24–7.20 μm.^6,31,38^ Green *et al.*^38^ demonstrated that UHMWPE particulates measuring 0.24 μm in length elicited the greatest rate of bone resorption and cytokine [tumor necrosis factor-α (TNF-α), interleukin (IL)-1β, IL-6, and prostaglandin E_2_ (PGE_2_)] production in an *in vitro* mouse model at a particulate volume (μm^3^) to macrophage ratio of 10:1. When increasing the ratio to 100:1 the response dissipated; however, particulates measuring 0.45 and 1.71 μm in length then became active, paralleling their findings in another similar study.^39^ The work of these respective authors argues that wear debris incites a unique immune response dependent on both particulate size and dose, likely incorporating natural phenomena of specificity, critical thresholds, overstimulation, and desensitization.

When challenged with wear debris, three general cellular responses have been described, depending on particulate size. Particles <150 nm are pinocytosed, particles 150 nm to 10 μm are phagocytosed, and particles >20 μm induce multinucleated giant cell formation, with some overlap.^6,7,31^ It should be noted that multinucleated giant cell formation and activity are often not analyzed *in vitro*, such as in the study by Green *et al.*^38^ described above, due to long incubation time and unique culture requirements. Taking this into account, it quickly becomes evident why larger particles elicited no immediate osteolytic reaction in that study. The overall immune response to UHMWPE particles has been characterized as a foreign body reaction, marked by granulomatous chronic inflammation.^7,31^

## Innate immune response

Although cells of monocytic lineage dominate in the pathogenesis of osteolysis, numerous cell types and inflammatory mediators have been implicated, summarized in Tables 1 and 2, respectively. Indicative of the innate immune system, NALP3 inflammasome, nuclear factor-κB (NF-κB), TNF-α, and IL-1β activation are significant to this response.^5–8,22,31^ Pathogenesis is diverse and multifactorial, but is ultimately driven by increased osteoclastogenesis, proinflammatory enzymatic bone resorption, and decreased osteoblastic activity. Although no secondary, adaptive immune response is implicated in UHMWPE wear-induced osteolysis, the innate response does occur in a sequential manner. The three main pathways favoring bone resorption mentioned above do not occur in isolation, but rather as a comprehensive process shifting normal bone metabolism in favor of catabolism, making a temporal presentation of osteolysis difficult. Therefore, pathogenesis will be presented in a component-based system, exhibiting temporal progression as appropriate.

T-lymphocytes have been found in periprosthetic tissue with osteolysis but are generally detected in low numbers.^40,41^ Furthermore, Pfp/Rag2 double-knockout mice (entirely lacking mature T and B lymphocytes with severe depletion of NK cell function) have been shown to develop osteolysis at similar rates to control mice with similar amounts of locally produced TNF-α and IL-1β.^41^ However, a role has been proposed for lymphocytes in osteolysis, specifically a hypersensitivity reaction to metal-on-metal prostheses.^6,7,9,40^ As this current study focuses on UHMWPE wear-induced osteolysis, these reactions are beyond the scope of this study. Therefore, lymphocytes and the adapative immune response will be excluded from any further discussion.

### Cellular response

**Figure 1:**
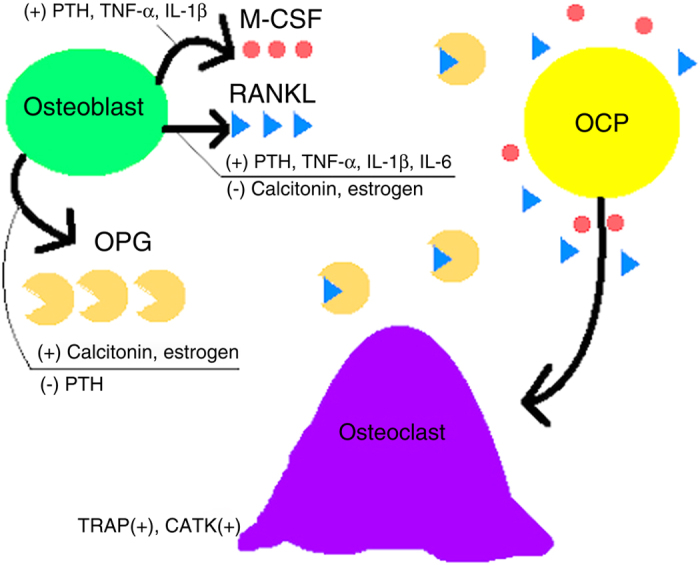
Hormonal regulation of osteoclastogenesis. Upon M-CSF and RANKL stimulation, OCPs undergo differentiation into giant, mature osteoclasts expressing TRAP and CATK.

In order to fully comprehend the roles of monocytes, dendritic cells (DC), osteoclasts, osteoblasts, and fibroblasts in osteolysis, a basic understanding of physiologic bone metabolism must be realized. Bone is constantly under revision, formed by osteoblasts and broken down by osteoclasts under hormonal and paracrine regulation. When this homeostatic process is misbalanced in favor of catabolism, osteolysis results. Briefly, osteoclastogenesis is mediated by two growth factors: receptor activator of NF-κB (RANK) ligand (RANKL) and macrophage colony-stimulating factor.^5,6,22,31,40,42^ RANKL is of the TNF superfamily; cytokines capable of inducing RANKL expression include TNF-α and IL-1β, among others. RANKL increases TNF-α production and TNF-α enhances NF-κB activation, forming a compounding and synergistic positive feedback loop. Stimulation of monocytes and osteoclast progenitors (OCP) by RANKL and macrophage colony-stimulating factor results in their fusion and differentiation into mature, multinucleated osteoclasts in tissue. Osteoblasts, which produce osteoprotegerin (OPG) or osteoclastogenesis inhibitory factor, are central to NF-κB regulation. OPG functions as an extracellular decoy receptor for RANKL, thereby inhibiting RANK stimulation and osteoclastogenesis. Finally, these factors, which influence osteoblast/clast genesis, function, and survival, are mainly controlled by the interplay between parathyroid hormone, calcitonin, and estrogen under normal physiological conditions, summarized in Figure 1. Once fully differentiated, mature osteoclasts express tartrate-resistant acid phosphatase (TRAP) and cathepsin K (CATK), two enzymes essential to their lytic function.

UHMWPE particles induce inflammation and stimulate macrophages by a variety of mechanisms. One such mechanism occurs through Toll-like receptor (TLR) activity. TLRs are expressed in many inflammatory cells, especially those of monocytic lineage, and their stimulation promotes phagocytosis and initiates an innate immune response mediated through NF-κB activation.^43–45^ Classically, TLRs are stimulated by pathogen- or damage-associated molecular patterns in response to infection or tissue damage, allowing for an early inflammatory response. Maitra *et al.*^46^ demonstrated that UHMWPE particles are comprised of alkane polymers capable of directly stimulating TLR2 and TLR1/2 *in vitro*, leading to their phagocytosis and subsequent immune activation. When recovered from osteolytic tissue, a majority of these alkane polymers were found to have undergone extensive carbonyl modification, indicative of oxidative damage likely mediated enzymatically by cells of monocytic lineage. A positive correlation was observed between amount of oxidation of alkane polymers and their affinity for TLR, with oxidized polymers exhibiting up to a 140-fold increase in binding affinity. In a subsequent study,^44^ Maitra *et al.* observed that the endosomes of inflammatory infiltrates of periprosthetic tissue challenged with UHMWPE were engorged with alkane particles. The failure of the endosomal enzymes to degrade UHMWPE led to an increase in endosome compartment size, number, and fusion, with some observed along the plasma membrane undergoing exocytosis. Unsurprisingly, necrotic cells were observed in the surrounding tissue, with UHMWPE particles, cathepsin, and collagen damage detected. This tissue injury generates heat shock proteins and other damage-associated molecular patterns,^7,47^ further increasing TLR activation. Incubation with oxidized alkanes activated DCs, macrophages, monocytes, and osteoclasts, with each expressing an upregulation of surface major histocompatibility complex class II, B7–1, B7–2, and CD40 molecules.^44^ Significantly, these cells exhibited an increase in TLR1 and TLR2 mRNA transcription, with the upregulation of IL-1, IL-6, IL-10, IL-12, TNF-α, and IFN-γ. These findings present many implications for the immune response to UHMWPE wear, most importantly that UHMWPE is capable of inciting a nonspecific and self-propagating innate immune response.^47^

**Figure 2:**
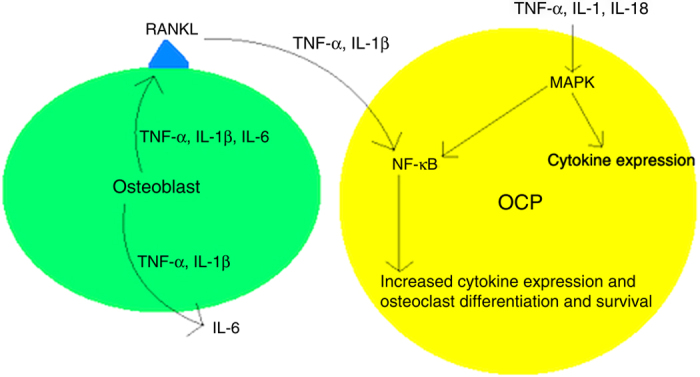
Effects of major cytokines involved in osteolysis. Note the repetitive and propagative properties of the immune response. RANKL, TNF-α, and IL-1β also inhibit osteoclast apoptosis.

Another important finding by Maitra *et al.*^44^ in the previously described study is that TLR stimulation of monocytes and macrophages induced pro-IL-1β expression but not the release of mature IL-1β, the actions of which are summarized in Table 2. Instead, endosomal destabilization and NALP3 inflammasome activity have been characterized as mediators of IL-1β and IL-18 maturation and release.^4,7,44,48–50^ NALP3, when activated, complexes with adapter protein PYCARD, leading to caspase-1 recruitment and activation. Caspase-1 subsequently cleaves pro-IL-1β and pro-IL-18 into their active forms, allowing for their secretion. DCs treated with UHMWPE particles *in vitro* expressed increased levels of caspase-1 and active IL-1β and IL-18, which were subsequently downregulated after cathepsin inhibition.^44^ In demonstrating that NALP3 inflammasome activity was integral to the development of pneumoconiosis (a disease process also characterized by foreign body reaction), Cassel *et al.*^50^ reported that inflammasome activation required reactive oxygen species generation and an efflux of potassium. NALP3 inflammasome activation therefore occurs by two mechanisms, both of which are consistent with osteolysis: reactive oxygen species and other danger signal (ATP, urate, and so on) generation,^7,48,50,51^ and cathepsin release.^7,44^ When osteoclasts, macrophages, and DCs phagocytose UHMWPE particles <10 μm, they are unable to degrade them, leading to endosomal instability and the subsequent spilling of cathepsin.^7,44^ This damage undoubtedly releases both self and neighboring intracellular contents that likely signal NALP3 activation further, paralleling the findings by Cassel *et al.*^50^ Furthermore, as UHMWPE particles are released secondary to endosomal instability, they are likely re-phagocytosed by other activated cells, leading to a perpetual inflammatory cascade in the local periprosthetic environment. When challenged by larger UHMWPE particles >20 μm, multinucleated giant cells are formed that undergo “frustrated phagocytosis,” where UHMWPE is trapped near the cell surface, activating NADPH oxidases.^7,51^ These oxidases generate reactive oxygen species contributing to NALP3 inflammasome activation. Coupled with TLR-induced cytokine transcription, NALP3 inflammasome and caspase-1 activity allow IL-1β and IL-18 to contribute to development of osteolysis, as illustrated in Figure 2. A take home point from NALP3 inflammasome activation and activity is again that a nonspecific, self-propagating immune response is initiated, destroying natural tissue architecture in a local environment. Despite the inflammatory response described, detection of osteolysis remains challenging, expounded upon further in Detection and diagnosis section.

Although bone destruction is mainly regulated by local proinflammatory factors in a periprosthetic microenvironment, systemic macrophage/OCP involvement is reported. Ren *et al.*^28^ continuously infused saline or UHMWPE particles into mice femora and injected reporter bioluminescent macrophages into tail veins. Upon noninvasive *in vivo* imaging, significantly increased reporter macrophages were detected in femora of mice infused with UHMWPE, and these macrophages were positively stained for TRAP and were associated with increased bone destruction. Wu *et al.*^29^ found that peripheral monocytes in patients with AL demonstrated an increased frequency of the CD14^+^CD16^+^ phenotype compared with controls. The CD14^+^CD16^+^ phenotype was then correlated with increased periprosthetic tissue macrophage infiltration and pathology score, plasma TNF-α and IL-1β levels, and inducible TNF-α and IL-1β production after stimulation with UHMWPE particles, paralleling findings of CD14^+^CD16^+^ monocytes in rheumatoid arthritis.^29^ Coupled with the findings that TNF-α can increase CD16 expression^52^ and precede CD16^+^ monocyte expansion,^29^ Wu *et al.* concluded that TNF-α and CD16 likely form an auto-amplification loop. Applied to the local, periprosthetic environment in osteolytic tissue, this relationship likely has significant implications. Yet again exemplifying self-propagation, cytokine and chemokine production lead to the increased systemic migration of OCP and a potent, proinflammatory macrophage phenotype, resulting in a positive feedback loop of bone destruction.

Sharing lineage with monocytes, DCs have a role in osteolysis pathogenesis paralleling that of their close relatives. Maitra *et al.*^42^ conducted an *in vivo* study assessing DC function in a calvaria UHMWPE-induced osteolytic Csf1r knockout mouse model (profoundly deficient in osteoclasts, tissue macrophages, and Langerhans cells) with compelling results. Csf1r^-/-^ mice challenged with UHMWPE particles developed statistically significant osteolysis vs controls, and demonstrated peripheral DC recruitment exhibiting CATK expression and multinucleated giant cell formation around UHMWPE particles. Although DCs have been shown to contribute to osteoclastogenesis through cytokine production,^7,42,45,46,53^ the findings of Maitra *et al.* suggest DCs may yet have a more direct role in bone metabolism. While it should be noted that the robust DC response seen by Maitra *et al.* may have been exaggerated *ex vacuo*, that is, due to a lack of normal monocytic or resident macrophage-derived osteoclastogenic function, DCs likely function in conjunction with these cells under normal physiological conditions. Indeed, in response to UHMWPE particles, DCs have been demonstrated to infiltrate periprosthetic tissue,^7,44^ support prosthetic synovial pseudomembrane formation,^7^ produce TNF- α, IFN-γ, and IL-1, IL-6, and IL-12,^44,46^ upregulate major histocompatibility complex class II expression,^44,46^ and phagocytose UHMWPE particles.^7,44^ Thus, DCs should be considered as extensions of monocytes in regards to function and possible therapeutic intervention.

### Cytokine response

Although poorly understood, molecular pathways and targets of osteolysis are emerging. NF-κB, described as the master regulator of immune response,^47^ stimulates osteoclastogenesis,^31^ and is activated by RANKL, the production of which is increased by TNF-α and IL-1β.^54^ Osteoclastogenesis has also been reported in isolated OCPs when treated with TNF-α. Although debated, direct TNF-α-induced osteoclastogenesis is likely mediated by a RANKL-dependent mechanism.^31^ Lam *et al.*^55^ cultured purified OCPs with TNF-α and macrophage colony-stimulating factor and found that the OCPs failed to undergo osteoclastogenesis. When cells recovered from marrow were exposed to RANKL, differentiation did occur, paralleling conflicting findings by other authors.^56,57^ Upon addition of OPG to initiation of bone marrow culture,^55^ this effect was abolished, suggesting RANKL-dependent TNF-α-induced osteoclastogenesis. When OCPs were treated with RANKL and TNF-α, TNF-α was found to augment osteoclast differentiation in a dose-dependent manner. This synergistic effect was so powerful that 1 ng·mL^−1^ of TNF-α was sufficient to potentiate osteoclast differentiation at RANKL doses <1% of that normally required for osteoclastogenesis.

Regarding mature osteoclasts, TNF-α activates osteoclasts independent of and similar in potency to RANKL, and also greatly augments RANKL-induced osteoclast activation.^58^ Fuller *et al.*^58^ treated osteoclasts harvested *in vitro* and *in vivo* with TNF-α and discovered a potent stimulation of actin ring formation (indicative of activation and bone resorptive capability) comparable to that induced by RANKL. When incubated with OPG, no inhibition of TNF-α-induced actin ring formation occurred, suggesting a RANKL-independent mechanism for osteoclast activation. Incubation with small amounts of TNF-α and RANKL exhibited a greatly synergistic response paralleling actin formation induced by RANKL alone at concentrations magnitudes higher. Lee *et al.*^59^ treated osteoclasts with various factors *in vitro* and found that TNF-α, IL-1β, and RANKL promoted osteoclast survival. TNF-α inhibited apoptosis the strongest, and this inhibition was dependent on Akt and ERK phosphorylation. By enhancing osteoclast differentiation, activation, and survival often in concert with RANKL, TNF-α significantly contributes to the development of osteolysis.

IL-1 serves to collaborate with TNF-α. Wei *et al.* demonstrated that TNF-α increases IL-1 and IL-1 receptor (IL-1R) type I expression by murine marrow stromal cells, and that osteoclastogenesis induced by TNF-α was reduced ~50% with IL-1R antagonism.^60^ Moreover, TNF-induced *RANKL* gene expression was blunted in IL-1R-deficient cells, an effect not seen in control cells treated with active vitamin D (known activator of *RANKL* gene). TNF-α and IL-1 effects were mediated by p38-associated mitogen-activated protein kinases (MAPK). In addition, Wei *et al.*^60^ incubated OCPs derived from wild-type and TNF receptor-deficient mice with and without RANKL to assess IL-1 function. No osteoclast differentiation occurred in cultures without RANKL. Contrastingly, cultures incubated with RANKL and increasing amounts of IL-1 demonstrated a dose-dependent differentiation of osteoclasts. TNF receptor knockout had no bearing on these results. Thus, in addition to mediating the effects of TNF-α, IL-1 is also capable of directly stimulating osteoclastogenesis in the presence of low levels of RANKL. IL-1R and IL-18R signaling have been reported to activate NF-κB and MAPK, paralleling these findings.^48^ A conclusion to be drawn is that while TNF-α, IL-1, and RANKL display cooperative synergism to promote osteoclast differentiation, they also maintain independence to mediate these proinflammatory effects. The result is a fail-safe mechanism in initiating an immune response, one that possesses the ability to sustain and propagate itself, as described in earlier sections.

## Detection and diagnosis

Detection of osteolysis at stages early enough to implement intervention is challenging. First, osteolysis is often asymptomatic or undiagnosed until the need for revision surgery.^4,8,17,20^ Traditional imaging frequently underestimates the true amount of periprosthetic bone loss^17^ and its sensitivity is dependent upon lesion location,^61,62^ while the widespread implementation of newer radiographic techniques poses challenges of cost-effectiveness and radiation exposure.^4^ In addition, metal artifact from arthroplasties has greatly hindered visualization of the periprosthetic interface and therefore diagnosis utilizing computed tomography (CT) and magnetic resonance imaging (MRI).^61^ High-resolution three-dimensional CT (3D-CT) has been developed to provide a more sensitive and accurate method than plain radiograph for measuring the size and progression of periprosthetic lesions in patients suspected of having osteolysis;^63–65^ however, the need for longitudinal studies underlines the significant radiation risk in the population at large.

In a cadaveric study, MRI has been reported to be 95% sensitive and 98% specific, with mean error of actual lesion volumes of 1.4±2.4 cm^3^.^61^ In addition, MRI has been demonstrated to be more effective than CT and plain film in detecting lesions ⩽3 cm, but less accurate than CT in measuring lesion volume.^62^ Multiple advances in imaging technique have greatly enhanced metal artifact reduction in MRI,^66^ making it a reasonable option for osteolysis/AL surveillance. However, visualization in MRI is still problematic, artifact reduction is variable, evaluation of the acetabulum is obscured by the convex surface of the acetabular implant,^66^ and difficulty in clinical correlation exists. Significantly, both MRI and 3D-CT cannot detect inflammation that occurs at the initial and early stages of osteolysis, leading to formation of osteolytic lesions.

There is compelling evidence that imaging techniques directly targeting inflammatory cells within osteolytic tissue could become great tools for the detection of particle-induced inflammation. In diagnosis of periprosthetic infection and AL after TJA, nuclear medicine techniques including bone scrintigraphy (triple phase bone scan), leukocyte scintigraphy (white blood cell scan or WBCS), and 18F-labeled fluorodeoxyglucose (FDG)-positron emission tomography (PET) have gained considerable attention.^67–74^ Triple phase bone scan, which is easily performed and accessible, has varying sensitivities and specificities in the literature, but is generally considered sensitive but not specific with an accuracy of 50%–70%.^67–69,72,74^ WBCS utilizes *in vitro* or *in vivo* labeling techniques to compare radiotracer uptake in periprosthetic tissue with set reference points generally in the marrow.^69,72,74^ While various combinations of techniques utilizing WBCS may demonstrate high sensitivity and specificity for infection (accuracy of over 80%), even being considered the gold standard by some, numerous pitfalls exist. WBCS is complex, expensive, time consuming, requires multiple visits to perform, dependent on patient marrow characteristics, and involves contact with blood products and reinjection (*in vitro*) or immunogenic antibodies (*in vivo*). Importantly, in regards to AL, WBCS mainly labels neutrophils, so is relatively insensitive to the largely macrophage-driven response.

FDG-PET, based off the premise of increased glucose metabolism in activated leukocytes, is advantageous in that it involves just one injection, has quick results, has high target to background ratio, and provides high resolution.^69,72,74^ As such, the focus of FDG-PET studies lay in the differentiation of periprosthetic infection from AL, as both can clinically present similarly but have vastly differing treatments.^67–74^ From high-quality reviews in the literature, sensitivities, specificities, and accuracies for diagnosing infection in THA are generally reported to be roughly 85%, 90%, and 90%, respectively.^69,70,73^ For TKA, these values are roughly 96%, 77%, and 83%.^69,73^ Variable values across the literature are attributable to divergent criteria for image interpretation and diagnosis,^67–74^ while the decreased specificity of TKA may be accounted for by nonspecific tracer uptake in the knee^69^ or intense tracer uptake attributable to capsulitis/synovitis from AL.^73^ Regarding THA, a group of researchers from the University Hospital of Aachen, Germany was able to achieve a sensitivity, specificity, and accuracy of 94%, 95%, and 95% in assessing 92 prostheses for periprosthetic infection versus AL.^68^ Their criterion for diagnosis was based off of five basic FDG uptake patterns. FDG uptake in periprosthetic soft tissue correlated with infection, while increased FDG uptake near the femoral neck and in the whole prosthesis-bone interface of the acetabular cup or in wide parts of the prosthesis-bone interface of the femoral stem correlated with AL. With these and further advancements, PET seems a promising tool to diagnose and potentially treat osteolysis and AL.

Utilizing *in vivo* imaging, the first report of systemic leukocyte recruitment in particle-induced inflammation was most likely published by Ren *et al.*^75^ They injected cement particles into femoral cavity of nude mice and then intravenously administered luciferase-expressing macrophages, demonstrating significant accumulation of macrophages at the femoral site with particles. The results of this study indicate that modulation of signaling mechanisms governing leukocyte recruitment may contribute to the treatment of osteolysis. On the basis of the activation of NF-κB in inflammatory cells stimulated by wear particles, Takahashi *et al.* described the feasibility of NF-κB/luciferase transgenic mice for *in vivo* imaging of inflammation and osteolysis in calvariae loaded with PE particles, highlighting a useful tool for evaluating the efficacy of treatments for particle-induced osteolysis.^76^

Recently, a polymeric nanocarrier system targeting inflammatory cells was revealed by a series of experiments performed by a group of researchers at the University of Nebraska, to be enriched at inflamed sites with a few animal models.^8,77–80^ The system makes use of water-soluble copolymers of *N*-(2-hydrocpropyl)methacrylamide (HPMA), that were originally designed for cancer chemotherapy due to the meritorious properties of the nanoparticles. These include an enhanced permeability and retention effect, water-solubility, low immunogenicity, and high stability.^81,82^ While HPMA copolymers have been labeled with near-infrared dye 800CW and radioisotope ^125^I for imaging purposes^78,80^ and loaded with a drug dexamethasone for therapeutic intention,^77,78^ it is anticipated that conjugates of these copolymers with other radioisotopes such as ^111^In, ^99m^Tc, ^18^F, and ^64^Cu, and other drugs, will be explored in the future in order to achieve the clinical use of nanocarriers for early diagnosis and effective treatment of periprosthetic osteolysis. These nanocarriers are in contrast to FDG-labeled probes, which are not feasible for drug conjugation as they are too nonspecific and lack durability.

**Figure 3:**
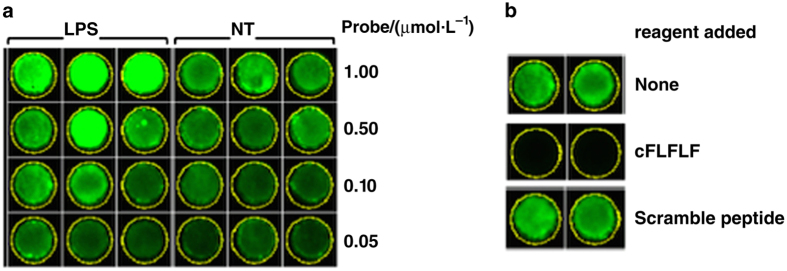
Binding assay of NIRF probe cFLFLF-PEG-Cy7 to RAW264.7 cells stimulated by 1 μg·mL^−1^ lipopolysaccharide vs no treatment (NT).^90^ (**a**) Various concentrations of the probe were applied to the assay, including (from top to bottom) 1.0, 0.5, 0.1 and 0.05 μmol·L^−1^. Results indicate that fluorescent intensity strengthened with increasing doses of the probe, and that LPS was able to enhance binding affinity of the probe for cells. (**b**) A blocking test was performed with cFLFLF and a Scramble peptide (cLFFFL), revealing that the binding signal was blocked by cFLFLF but not controls.

Formyl peptide receptors are a group of proteins within the G-protein-coupled receptor family, and are primarily expressed on the surface of activated leukocytes.^83^ Utilizing cinnamoyl-Phe-(D)-Leu-Phe-(D)-Leu-Phe-Lys (cFLFLF), we have developed an innovative platform technology for a series of tracers detecting inflammation, with modular designs including leukocyte recognition (antagonistic, synthetic peptide with high affinity), optimization of pharmacological properties (linker), and a molecular imaging module for appropriate label detection. Utilization of cFLFLF is based on the fact that it is a well-defined, non-natural amino acid-containing synthetic peptide with high affinity for formyl peptide receptors, and has small molecular weight, water-soluble, and functionally inactive properties. The cFLFLF-PEG modules have been successfully utilized by us and others,^84,85^ for building PET (DOTA-^64^Cu),^86^ optical imaging (Cy5 and Cy7),^87,88^ and MRI (DOTA-Gd)^89^ probes, and have exhibited excellent imaging in a variety of inflammation models in animals. Noticeably, an *in vitro* experiment showed that the cFLFLF-PEG-Cy7 probe has specific binding affinity to the cultured, murine macrophage cell line RAW264.7 stimulated by lipopolysaccharide (Figure 3).^90^ Furthermore, the cFLFLF-PEG-^64^Cu probe has been employed to target macrophage activity in apolipoprotein E-deficient (ApoE−/−) mice with type 2 diabetes mellitus,^91^ and in a chemically-induced osteoarthritic rat model.^90^ Our next plan is to explore the feasibility of utilizing cFLFLF-PEG as a carrier of various imaging and therapeutic agents for animal models of periprosthetic osteolysis, with the ultimate goal of developing these agents for the early diagnosis and treatment of osteolysis in improving clinical outcomes.

The clinical implementation of *in vivo* imaging techniques provides diverse and unique challenges compared with other imaging modalities. Many of these challenges are related to the probes themselves. Probes should have high and rapid uptake, selectivity, the ability to reside in target tissues long enough to allow optimal imaging, efficient clearance to improve target to non-target ratio, and the absence of adverse effects. In addition, probes should have low radiation or toxicity, ease of accessibility, long shelf life, ease of use, and acceptable cost. Finally, like other imaging techniques, standardizations for diagnosis and therefore efficacy of these modules must be attained. Keeping these in mind, it quickly becomes evident why the synthesis of probes acceptable and feasible for human use is such a difficult task.

## Treatment

Medical treatment of osteolysis has faced many challenges to date: detection of disease at stages early enough to implement efficacious intervention, quantitative outcome measure of treatment efficacy, and either safe or targeted delivery of drugs with potentially devastating systemic side effects.^8,21^ Agents studied for treatment of osteolysis are those utilized in other catabolic bone diseases, namely bisphosphonates,^9,21^ corticosteroids,^5,8^ TNF-α antagonists,^5,9,21^ IL-1 antagonists,^5,21^ IL-10 vectors,^5,31^ and OPG vectors.^5,31^ Bisphosphonates have proven unsuccessful in inflammatory conditions and clinically are generally not supported by the literature,^92^ while systemic corticosteroids are avoided due to unwanted and morbid side effects.^5^ Purdue *et al.*^8^ linked dexamethasone to an HPMA copolymer and found that single dose tail vein injection into an osteolytic mouse model completely prevented osteolysis, paralleling findings of a preliminary study in an arthritic rat model where arthritis was prevented for over four weeks.^77^ In additional experiments, Purdue *et al.*^8^ found that this sustained effect of drugs coupled to HPMA copolymers was due to their rapid, nonspecific endocytic uptake and sequestration by inflammatory cells at sites of inflammation, the benefits of which are extremely advantageous: HPMA copolymers are capable of quick localization with high selectivity and demonstrate high durability. TNF-α and IL-1 antagonists have variably demonstrated efficacy, but come with unwanted immunosuppression.^5,21^ Anti-cytokine (*IL-10* and *OPG*) gene therapy has demonstrated promising results in animal models, but reluctance to implement viral gene therapy in humans still exists.^5^ In addition, adenoviral vectors are commonly used, which have high immunogenicity and are warned against.^5^ As *in vitro* gene transfer and subsequent transplantation into human subjects remains a distant possibility, *in vivo* viral gene therapy likely is not a reasonable or realistic solution, especially as osteolysis is a chronic disease process.

Recently, targeting RANK/RANKL has emerged as a potential therapeutic avenue. Denosumab (Amgen; Thousand Oaks, CA, USA), a monoclonal antibody against RANKL, has recently been approved for use in bony metastasis, hypercalcemia of malignancy recalcitrant to bisphosphonates, and certain giant cell tumors. It is currently in phase II clinical trials regarding efficacy in osteolysis.^93^ Denosumab is mostly effective due to its anti-resorptive effects, as bone alkaline phosphatase did not decrease until a month after injection, while bone turnover markers immediately decreased.^9^ The finding that a human null mutation of RANKL results in osteopetrosis but not immunosuppression provides support for RANKL antagonism as therapeutic intervention, despite the central role of NF-κB in immune response.^94^

Additional support for targeting RANK/RANKL arrives from studies demonstrating inhibition of osteolysis by: treatment with methotrexate,^95^ ursolic acid,^96^ and triptolide^97^ (RANKL-mediated), p38 suppression^98^ (downstream signaling cascade induced by RANKL), and RANK small interfering RNA local injection.^99^ Methotrexate is an interesting option in that its effects are mainly mediated by the adenosine A_2A_ receptor, which has many downstream effects.^95^ In an *in vivo*, UHMWPE particle-induced osteolytic murine model, Mediero *et al.*^95^ found that methotrexate treatment increased alkaline phosphatase^+^ cells, OPG expression, and bone formation and density, and reduced RANKL expression, RANK/RANKL+ cells, inflammatory infiltrate, TRAP^+^ and CATK^+^ osteoclasts, and osteolysis. Considering the lower risk for failure by AL in rheumatoid arthritis patients,^100^ many of whom are likely on methotrexate, adenosine A_2A_ receptor agonism may provide an invaluable therapeutic option in osteolysis. Systemic side effects with methotrexate or adenosine receptor treatment again validate the utility of controlled, localized treatment, which are possible with newer *in vivo* imaging techniques.

## Perspective

Despite advances in material design of prostheses used in TJA, medical treatments must continue to be pursued due to the lack of long-term clinical data of newer prostheses and the plethora of arthroplasties already performed with those more classical. The increasing number of surgeries being performed on increasingly younger patients expounds this need for medical intervention. Inflammatory cells of monocytic lineage dominate pathogenesis of osteolysis, and therefore are the natural target for chemotherapy. Historically, the main barriers to implementation of effective therapy have been detecting disease at stages early enough for effective intervention and averting unwanted and significant systemic side effects. In theory, this can be accomplished by one of two methods: designing a drug without significant systemic side effects, or navigating a delivery mechanism that allows for localized and selective drug dissemination without systemic side effects. For example, a RANKL antagonist is currently in clinical trials for use in osteolysis, and this shows some promise in that it impacts immunocompetence less than originally thought. More importantly, copolymers utilized in newer *in vivo* imaging techniques have demonstrated excellent utility in detecting osteolysis early and, through linkers, providing selective and localized treatment able to be followed over time. HPMA and our cFLFLF modules demonstrate promising examples. With high selectivity, drugs targeting any significant pathway discussed may prove beneficial in the treatment of osteolysis, including RANK, NALP3 inflammasome, TNF-α, or caspase-1 antagonism, among others. As osteolysis develops over a period of 5–10 years, it may be reasonable to utilize *in vivo* imaging at post-operative intervals in the order of years, first screened at 5 years post-operatively. With early detection and selective therapy without significant side effects, osteolysis can be managed medically to decrease morbidity and improve outcome after TJA.

## Figures and Tables

**Table 1:** Role of essential cells implicated in osteolysis pathogenesis

Cell type	Role	Comments
Monocyte/macrophage	Dominant cell type implicated in osteolysis: vastly present in periprosthetic tissue and pseudosynovial membrane, and correlated with bone resorption^5–8,22,28–31^ Secrete MMPs, TNF-α, IL-1β, IL-6, and eicosanoids after phagocytosis of wear debris^101^ Systemic migration contributes to local infiltration, osteoclast differentiation, and bone destruction^28^ Increased peripheral CD14^+^CD16^+^ phenotype frequency; correlated with increased baseline level and production of TNF-α and IL-1β^52^	Potent, proinflammatory phenotype
Dendritic cell	Infiltrate and surround UHMWPE particles, participating in phagocytosis and MGC formation^7,42,44^ Produce proinflammatory cytokines IL-1, IL-6, IL-12, TNF-α, and IFN-γ ^7,42,44,46^ Compose prosthetic synovial pseudomembrane^7^	Ultimately results in NALP3 inflammasome activation and enzymatic bone resorption; likely paralleling macrophage function Contributes to osteoclastogenesis and activation
Osteoblast	Able to phagocytose wear particles and alter cellular signaling^6,102^ Decrease OPG secretion when challenged with PE *in vitro*^103^ Express RANKL^104^ and M-CSF^105^ upon TNF-α and IL-1β stimulation *in vitro* Dose-dependent decrease in proliferation, differentiation, and mineralization when challenged with UHMWPE particles *in vitro*^102^ Decreased expression of procollagen α1 mRNA and synthesis of collagen I *in vitro* by phagocytic- dependent and -independent mechanisms when challenged with PE particles^101^ Increased expression of IL-6^101^ and IL-8^103^ when challenged with PE *in vitro*	
Fibroblast	Present in pseudosynovial periprosthetic membrane^9,40^ Expresses RANKL and can induce osteoclast differentiation in pseudosynovial membrane^106^ Activated by TNF-α and IL-1β and produce MMPs and cytokines in titanium wear^107^ and osteoarthritis^108^ models In tissues exposed to PE, possess MMP2 mRNA alongside macrophages and MGCs expressing MMPs 2 and 14 protein but only MMP14 mRNA^109^	Likely activated by TNF-α and IL-1β in presence of PE as well, contributing to inflammation Suggests cooperation with macrophages in local tissue destruction

IL-1, interleukin-1; IFN-γ, interferon-γ; M-CSF, macrophage colony-stimulating factor; MGC, multinucleated giant cell; MMP, matrix metalloproteinase; OPG, osteoprotegerin; PE, polyethylene; RANKL, receptor activator of nuclear factor-κB ligand; TNF-α, tumor necrosis factor-α; UHMWPE, ultra-high molecular weight polyethylene.

**Table 2:** Role of key inflammatory mediators contributing to osteolysis

Molecular effector	Role	Comments
TNF-α	Increases RANKL expression^54^ Strongly augments RANKL-induced osteoclastogenesis^55^ Independently activates osteoclasts with similar potency of RANKL; augments RANKL-induced activation^58^ Inhibits osteoclast apoptosis by Akt and ERK phosphorylation^59^ Strongly suppresses procollagen 1 expression^101^ Increases IL-1 and IL-1R type I expression *in vitro*^60^ Enhances macrophage-attractant chemokine production^110^	
IL-1	Increases RANKL expression^54^ Inhibits osteoclast apoptosis^59^ Mediates TNF-α-induced RANKL expression^60^ Enhances osteoclastogenesis in presence of RANKL^60^ Capable of activating MAPK and NF-κB^48^	
IL-6	Secreted by osteoblasts in response to wear particle, IL-1β, and TNF-α stimulation^6^ Secretion induced by NF-κB activation^7^ Released by stimulated macrophages and associated with increased osteolysis^7,31^ Stimulates RANKL expression on osteoblast cell surface in inflammatory state^111^	
IL-18	Activates MAPK^48^	
RANKL	Inhibits osteoclast apoptosis^59^	
NALP3 inflammasome	Activated by cathepsin,^7,44^ and ROS, in conjunction with other intracellular danger signals (ATP, K^+^, urate, etc.)^7,48,50,51^ Complexes with PYCARD, leading to caspase-1 recruitment and activation^4,7,44,48–50^	Activation occurs through nonspecific danger signals
Caspase-1	Regulator of inflammation and cell survival and differentiation^48^ Cleaves pro-IL-1β and pro-IL-18 into active forms, allowing for their secretion^4,7,44,48–50^ Increased levels, along with IL-1β and IL-18, after *in vitro* treatment of DCs with UHMWPE; this response diminished with cathepsin inhibition^44^	
TLR	Activated by DAMPs released in tissue damage^7,47^ TLR2 and 1/2 activated by UHMWPE alkane polymers with a 12–16 carbon length^46^ Activation by alkane polymers enhanced by polymer oxidative damage up to 140-fold^46^ Stimulation ultimately leads to DC, monocyte, macrophage, and osteoclast activation, with upregulation of MHC-II, B7–1, B7–2, CD40, IL-1, IL-6, IL-10, IL-12, TNF- α, and IFN-γ, as well as TLR1 and 2^44^ TLRs 2, 4, 5, and 9 observed in monocytes/macrophages of osteolytic tissue *in vitro*, with TLR2 and 5 response dominant^45^ TLR9 characterized as strongest promoter of phagocytosis in a bacterial model^43^	Taken together, these first four points demonstrate a nonspecific and self-propagating immune response, indicative of the innate immune system
Complement	PE activates alternative complement pathway, likely through Factor B^112^ Complement factors adsorbed to PE particles after activation^112^ Complement activation enhances vascular permeability, chemotaxis, and phagocytosis^112^ C3 demonstrates ability to recruit osteoclasts,^113^ and activate NF-κB^40^	Along with TLR, represents early, nonspecific immune response
Matrix metalloproteinase (MMP)	MMPs 1, 9, 10, 12, and 13 highly elevated in AL periprosthetic tissue, in addition to lesser elevation of others^114^ MMPs 1, 2, 3, and 9 identified in macrophages, fibroblasts, and endothelial cells of AL periprosthetic tissue^115^	Combined action capable of degrading almost all elements of periprosthetic extracellular matrix^114^
MAPK	Activated by TNF-α, IL-1, and IL-18^48,60^ Signaling leads to IL-1 and other cytokine expression, as well as NF-κB activation^48,60^	

DAMP, damage-associated molecular pattern; DC, dendritic cell; IL-1, interleukin-1; IFN-γ, interferon-γ; MHC-II, major histocompatibility complex class II; PE, polyethylene; RANKL, receptor activator of nuclear factor-κB ligand; ROS, reactive oxygen species; TLR, Toll-like receptor; TNF-α, tumor necrosis factor-α; UHMWPE, ultra-high molecular weight polyethylene.
